# Current status and future perspective of linked color imaging for gastric cancer screening: a literature review

**DOI:** 10.1007/s00535-022-01934-z

**Published:** 2022-10-26

**Authors:** Kazuo Yashima, Takumi Onoyama, Hiroki Kurumi, Yohei Takeda, Akira Yoshida, Koichiro Kawaguchi, Naoyuki Yamaguchi, Hajime Isomoto

**Affiliations:** 1 Division of Gastroenterology and Nephrology, Faculty of Medicine, Tottori University, 36-1 Nishicho, Yonago, 683-8504 Japan; 2 Department of Endoscopy, Nagasaki University Hospital, Nagasaki, Japan

**Keywords:** Linked color imaging, Image-enhanced endoscopy, Early gastric cancer, Gastric cancer screening, *Helicobacter pylori*

## Abstract

Screening endoscopy has advanced to facilitate improvements in the detection and prognosis of gastric cancer. However, most early gastric cancers (EGCs) have subtle morphological or color features that are difficult to detect by white-light imaging (WLI); thus, even well-trained endoscopists can miss EGC when using this conventional endoscopic approach. This review summarizes the current and future status of linked color imaging (LCI), a new image-enhancing endoscopy (IEE) method, for gastric screening. LCI has been shown to produce bright images even at a distant view and provide excellent visibility of gastric cancer due to high color contrast relative to the surrounding tissue. LCI delineates EGC as orange-red and intestinal metaplasia as purple, regardless of a history of *Helicobacter pylori* (*Hp*) eradication, and contributes to the detection of superficial EGC. Moreover, LCI assists in the determination of *Hp* infection status, which is closely related to the risk of developing gastric cancer. Transnasal endoscopy (ultra-thin) using LCI is also useful for identifying gastric neoplastic lesions. Recently, several prospective studies have demonstrated that LCI has a higher detection ratio for gastric cancer than WLI. We believe that LCI should be used in routine upper gastrointestinal endoscopies.

## Introduction

Gastric cancer is the fifth most common cancer and the fourth leading cause of cancer-related deaths worldwide [1]. In Japan, the number of gastric cancer cases ranks second, and the number of deaths caused by gastric cancer ranks third [2], making it a critical public health problem. It is widely recognized that *Helicobacter pylori* (*Hp*) is a carcinogen for gastric cancer [3, 4], with numerous studies reporting the development of atrophic gastritis and intestinal metaplasia following *Hp* gastritis, ultimately leading to gastric cancer [5, 6]. Eradication therapy is popular based on the evidence that *Hp* eradication reduces the development of gastric cancer and other diseases, such as peptic ulcers. As a result, conventional *Hp*-related gastric cancer cases are decreasing; however, post-*Hp*-eradicated and *Hp*-uninfected gastric cancers are increasing [7].

To ensure a good prognosis, it is essential to detect and treat gastric cancer at an early stage during screening. Endoscopic mucosal resection or endoscopic submucosal dissection are commonly indicated for early gastric cancer (EGC) where lymph node metastasis is negligible, because of their minimal invasiveness and potential for organ preservation. These treatments ensure high healing potential and maintain a patient's quality of life after treatment [8]. However, flat gastric cancer, the most common type of EGC, is often difficult to detect with an endoscope, even by well-trained endoscopists, because it has subtle morphological or color features and mixed presentations with atrophic gastritis and intestinal metaplasia [9–12].

In recent years, advances in image-enhancing endoscopy (IEE) have improved the ability to detect and diagnose early-stage gastric cancer. IEE methods include narrow-band imaging (NBI), blue laser imaging (BLI), and linked color imaging (LCI) [13]. In clinical practice, most of these methods must be combined with a magnifying endoscope to observe the structures and blood vessels of the gastric mucosal surface. The effectiveness of IEE using magnifying endoscopes has been widely reported for EGC diagnosis. However, only a few studies have reported the benefits of IEE without magnification. This is probably due to the low brightness of IEE, which prevents clear observation of the gastric mucosa at a distant view. Therefore, there is a need for new screening methods for early-stage gastric cancers. In this regard, LCI has been shown to have sufficient brightness for mid-to-long-range observation and provides excellent visibility of gastric cancer with a high color contrast relative to the surrounding tissue [13, 14]. Using LCI, it has become possible to diagnose inflammatory changes that are difficult to distinguish from cancer. Several case reports and studies have reported that LCI can more easily identify early-stage gastric cancer than the conventional white-light imaging (WLI) technique [13, 14]. This has been further supported by recently published prospective studies, which have demonstrated that LCI has a higher detection ratio for gastric cancer than WLI [15–17]. The aim of this review was to summarize the current and future status of LCI as a diagnostic imaging method and describe its usefulness and challenges in clinical practice.

## EGC diagnosis by IEE

While chromoendoscopy using indigo carmine dye to produce contrast was previously commonplace, new IEE methods, such as NBI and BLI, are now becoming widely accepted in clinical practice. Both NBI and BLI have been reported to be useful in the qualitative diagnosis of EGC when combined with magnifying endoscopy [18–24]. The magnifying endoscopy simple diagnostic algorithm for early gastric cancer (MESDA-G) has been proposed as an integrated diagnostic system to identify EGCs by assessing the boundaries between lesions and healthy tissue, and to detect irregularities in microvascular and microsurface patterns in the gastric mucosa [25]. However, this technique has been associated with limitations: its use in wide lumen organs, such as the stomach, results in weak images at a distant view and insufficient visualization of the mucosal microstructure of the tumor surface [26]. In contrast, LCI, developed by Fuji Film Co., Ltd, has been reported as an unprecedented and revolutionary screening diagnostic method that provides sufficient brightness and color differences, even in wide lumens [13, 14].

## Characteristics of LCI

In 2012, the LASEREO system, which uses LASER as light source, was developed by and made commercially available from Fuji Film Co., Ltd, along with BLI. In 2014, LCI was also developed. Laser light, with a short wavelength of 410 nm, reaches only a very short distance from the mucosal surface layer and is specifically absorbed by hemoglobin. This characteristic is used to clearly depict mucosal surface blood vessels and to depict the difference in mucosal surface tissue structure, including surface blood vessels, as color differences [13, 14].

BLI images are created by cutting the red component of the lighting during post-processing. In LCI, brightness and color contrast are enhanced by the red component during post-processing. The brightness of LCI makes it suitable to be used for distant views, allowing red areas to appear more vividly red and white areas to appear whiter, thereby enabling endoscopists to identify suspicious lesions more easily. Providing images with enhanced color differences is a key feature of LCI as it can help differentiate suspicious lesions from that of the surrounding mucosa by color. It has been reported that the demarcation of lesions in LCI mode is clearer than that in WLI mode [27].

Following the development of endoscopic systems using laser, endoscopic systems using light-emitting diodes (LEDs) (ELUXEO ENDOSCOPIC SYSTEMS; Fuji Film Co., Ltd, Tokyo, Japan) were released in 2020. The ELUXEO system was developed to precisely control the emission intensity ratio of LED illumination in the four colors of blue-violet, blue, green, and red, generate white light and short-wavelength narrow-band light, and to obtain high-quality images equivalent to LASEREO. LED-LCI is expected to have the same diagnostic performance as conventional LCI (Laser-LCI) [28, 29]. LCI can be said to be a new diagnostic method for gastric cancer screening that utilizes higher color contrast images to distinguish tumors from the surrounding mucosa.

## LCI for the diagnosis of *Hp* gastritis

**Fig. 1:**
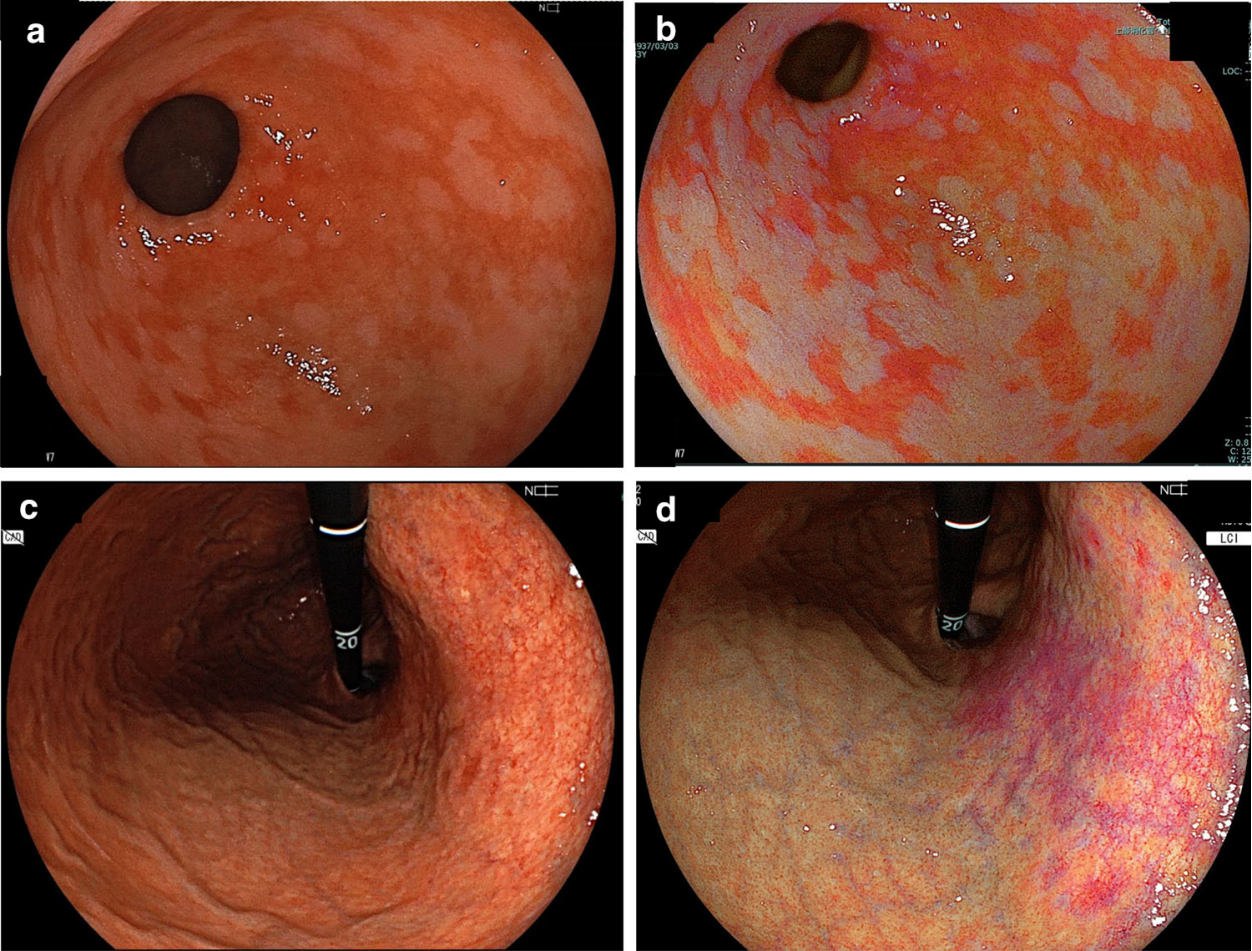
Representative endoscopic images of *H. pylori*-associated gastritis using white-light imaging (WLI) and linked color imaging (LCI). **a** WLI image of intestinal metaplasia in the antrum. **b** LCI image of intestinal metaplasia in the antrum (lavender color). **c** WLI image of mucosal atrophy and map-like redness in the gastric body. **d** LCI image of mucosal atrophy and map-like redness in the gastric body

Gastric mucosal inflammation (chronic gastritis), severe atrophy, and intestinal metaplasia caused by *Hp* are generally considered risk factors for gastric cancer [30–32]. The Kyoto classification of gastritis has clarified the endoscopic findings for assessing *Hp* infection status and identified the risk factors for EGC [33–35]. Ash-colored nodular changes are endoscopic signs of intestinal metaplasia detected using WLI. Despite the high risk of gastric cancer, it is difficult to diagnose gastrointestinal metaplasia in chronic gastritis [36–39]. Existing data show that gastrointestinal metaplasia is seen as purple (lavender color) mucosa on LCI and green mucosa with patchy distributions on BLI (Fig. 1a, b) [22, 36, 40]. If it cannot determined whether the color of the mucosa is pale purple or another color on LCI, BLI may be used to identify green mucosa, which strongly suggests intestinal metaplasia [22, 36].

LCI has also been considered useful for evaluating gastritis according to the Kyoto classification of gastritis [41–43]. On LCI, diffuse redness of the gastric mucosa of the gastric body, which is a classic feature of current *Hp* infection, is observed clearly as deep red (crimson) in color, *Hp*-negative mucosa after eradication is observed clearly as apricot in color, and intestinal metaplasia appears clearly as lavender in color. Due to these distinctive color differences, it has been reported that the detection rate of LCI for intestinal metaplasia is significantly higher than that of WLI [44–46]. Dohi et al. [44] reported that LCI improved the endoscopic diagnosis of active *Hp* infections, with 10–15% improvements noted in accuracy, sensitivity, and specificity, when compared with WLI. In a multicenter prospective study by Ono et al. [8] comparing the accuracy of WLI and LCI for the endoscopic diagnosis of *Hp* gastritis, LCI was significantly more accurate than WLI in patients with past infections. Another study by Ono et al. [42] also showed that the lavender color in LCI allows for the non-invasive detection of gastrointestinal metaplasia. Recently, a meta-analysis showed that LCI has good diagnostic efficacy for gastrointestinal metaplasia [47]. Furthermore, when observed with LCI, the vascular permeation of the atrophic border and the atrophy region becomes clearer (Fig. 1c, d) than when observed with WLI [48]. Majima et al. [49] reported that the positive risk finding of map-like redness (MR) (Fig. 1, d) and the negative risk finding of regular arrangement of collecting venules for EGC detection after *Hp* eradication is more frequently detected by LCI than WLI. Recently, Ishida et al. [28] concluded that LED endoscopy is equivalent to laser endoscopy for assessing *Hp* infection status [28]. In addition, LED-LCI was more effective than WLI for evaluating *Hp*-related gastritis. Therefore, LCI appears to play an important role in determining the status of *Hp* infections.

## LCI for screening of EGC

**Fig. 2:**
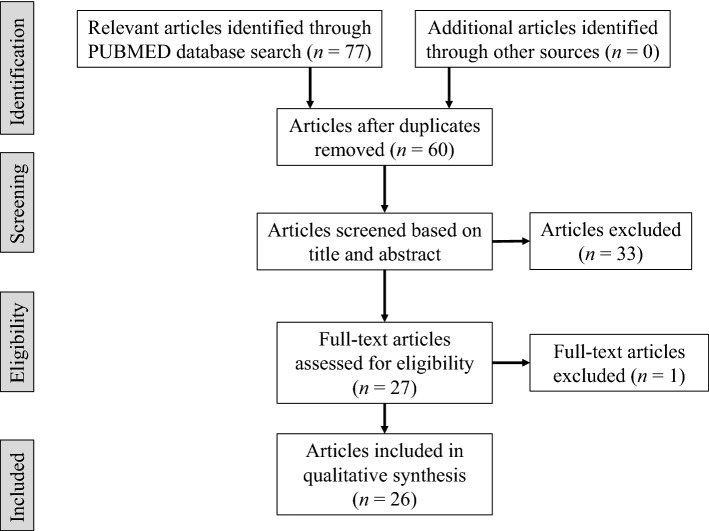
PRISMA 2009 flow diagram describing the selection of the studies included in our review

Most gastric cancers occur after chronic mucosal inflammation, especially intestinal metaplasia, due to *Hp* infection. In such background mucosa, early detection of gastric cancer is difficult even for well-trained endoscopists, and EGCs are often missed during screening endoscopy using WLI alone [9–12]. Table 1 shows a summary of studies on LCI for the detection of gastric neoplasms [15–17, 27, 28, 46, 50–69]. To prepare Table 1, we identified relevant studies in the literature by searching the databases of PubMed (Fig. 2). The search was restricted to the period from August 2013 to July 2022 and focused on the articles that were published in English on the detection of gastric cancer via LCI. The following search terms were used: (1) (Linked color imaging gastric cancer) AND (“2013/08/01”[Date—Publication]: “2022/07/31”[Date—Publication]; (2) (Linked color imaging upper gastrointestinal neoplasm) AND [(“2013/08/01”[Date—Publication]: “2022/07/31”[Date—Publication]. We also reviewed the reference lists of the selected studies to manually identify further relevant studies. Articles were excluded from the search if (1) the article was a review, basic research, editorial, or commentary; (2) the study had insufficient information and descriptions; and (3) the full text was unavailable. A total of 77 studies met the criteria. From these, 17 duplicate reports were excluded. Further, 8 review articles, a systematic review/meta-analysis, and an editorial were excluded as they were published in languages other than English. From the remaining 49 studies, 5 studies unrelated to gastric neoplasms were further excluded. Additionally, 14 studies regarding the evaluation of gastritis, a study regarding the evaluation of depth of EGC invasion, a case series regarding reflex esophagitis, a case report regarding gastric MALToma, and a study regarding the pathological evaluation of HER2 were excluded. Finally, 26 studies describing the detection of gastric cancer and upper gastrointestinal neoplasm, including gastric cancer, with LCI were selected (Table 1).

**Table 1:** Studies on LCI for gastric neoplasm detection

Author	Year	Study design	No. of subjects	Evaluation/Assessment	Findings/Outcome
Fukuda et al. [27]	2015	Case report	1	Real-time observation	LCI detected flat EGCs and clearly demonstrated the demarcation line between the malignant lesion and the surrounding mucosa without magnification
Ono et al. [50]	2017	Case report	1	Real-time observation	Gastric cancer in IM was observed as lavender in color on LCI
Kono et al. [51]	2018	Case report	1	Real-time observation	Combination use of AIM and LCI was useful for detection of EGC
Kubo et al. [52]	2019	Case report	1	Real-time observation	On LCI, flat EGC was highlighted as orangish, surrounded by lavender-colored tissue
Sun et al. [53]	2017	Retrospective/SC	62	Images of distal gastric diseases	LCI was a feasible and effective method for observing mucosal color changes regardless of the experience level of the endoscopist
Kanzaki et al. [54]	2017	Retrospective/MC	43	Images of EGCs and surrounding mucosa	The color difference between EGC and surrounding mucosa was significantly higher with LCI than with WLI
Yoshifuku et al. [55]	2017	Retrospective/SC	82	Images of EGCs	LCI improved the visibility of EGC, regardless of the experience level of the endoscopist or *Hp* eradication
Kitagawa et al. [56]	2018	Retrospective/SC	100	Images of small depressed gastric lesions	M-Chromo-LCI had significantly higher diagnostic accuracy than M-BLI (79.7% vs. 69.3%; *P* = 0.005)
Kitagawa et al. [57]	2019	Retrospective/SC	100	Images of gastric mucosal cancers ≤ 20 mm in diameter	The visibility score was significantly higher for LCI compared with WLI and BLI-bright (*P* < 0.001)
Fukuda et al. [46]	2019	Prospective/SC	52	Images of EGCs and surrounding mucosa	LCI images had higher color contrast between EGCs and surrounding mucosa compared with WLI
Fujiyoshi et al. [58]	2019	Retrospective/SC	43	Images of EGCs and surrounding mucosa	LCI was superior for EGC recognition (*P* < 0.0001), and the color difference between EGC and surrounding mucosa was significantly greater with LCI than WLI (*P* < 0.0001)
Kanzaki et al. [59]	2020	Retrospective/SC	61	Images of EGCs and suspicious mucosal areas	Based on color difference, EGC was not only reddish but also mixed with yellow, with an orange-like color on LCI
Sun et al. [60]	2020	Retrospective/SC	38	Images of EGCs and surrounding mucosa	RGB pixel brightness was useful and more objective in distinguishing EGC for LCI images, regardless of *Hp* infection status
Dohi et al. [61]	2020	Case report (Video article)	2	Real-time observation	LCI followed by M-BLI identified EGC in MR after successful *Hp* eradication with greater confidence than WLI in clinical practice
Kitagawa et al. [62]	2020	Retrospective/SC	154	Images and videos of EGCs after *Hp* eradication	LCI significantly improved the visibility and color difference of EGCs after *Hp* eradication (*P* < 0.001) and reduced miss rates (30.7% vs 64.9%, *P* < 0.001) compared with WLI
Yasuda et al. [63]	2021	Retrospective/SC	87	Images of EGCs and surrounding mucosa	LCI was more effective than IC and BLI-bright in the visibility of EGCs, especially for post-*Hp* eradication cases and flat or depressed lesions
Gao et al.[17]	2021	RCT/MC	2383 (high-risk population)	Real-time observation	The detection rate of gastric neoplastic lesions in a high-risk population was 4.31% in the WLI group and 8.01% in the LCI + WLI group (*P* < 0.001)
Matsumura et al. [64]	2021	Prospective/MC	31	Videos of EGCs	LCI had the highest visibility of EGCs after *Hp* eradication compared with WLI and BLI-bright
Yamaoka et al. [16]	2021	Prospective/SC	500 (patients with atrophic gastritis)	Real-time observation (WLI, followed by the LCI and BLI-bright)	75% of EGCs and gastric adenomas were detected by the first WLI mode; 25% of those were missed by the first WLI mode and were detected by the LCI mode or BLI-bright mode
Ono et al.[15]	2021	RCT/MC	1502 (patients with a history of gastrointestinal cancer)	Real-time observation	The detection rate of upper gastrointestinal neoplasms was higher with LCI (8.0%) than with WLI (4.8%); risk ratio, 1.67 [CI 1.12–2.50; *P* = 0.011]. The proportion of overlooked neoplasms was lower in the LCI group (0.67%) than in the WLI group (3.5%); risk ratio, 0.19 [CI 0.07–0.50]
Hiraoka et al. [65]	2021	Retrospective/SC	40	Images of EGCs and surrounding mucosa	The mean visibility scores for LCI with C2 enhancement were significantly higher than those with C1 enhancement. LCI with C2 enhancement tended to produce a color difference between the malignant lesions and the surrounding mucosa
Fockens et al. [66]	2022	Retrospective/MC	40 EGC lesions	Images of EGCs	Experts reached higher consensus on discrimination between neoplasia and inflammation when using LCI. Non-experts placed the biopsy mark more accurately with LCI (82.3% vs. 87.2%, *P* < 0.001). Non-experts were indicated to prefer LCI over WLI
Khurelbaatar et al. [67]	2022	Retrospective/SC	508 EGCs from 456 patients	Images of EGCs	LCI significantly improved visibility of EGC regardless of differences in lesion morphology, histology, location, depth of invasion, and *Hp* status compared with conventional WLI
Ishida et al. [28]	2022	Retrospective/SC	99 EGC lesions from 88 patients	Videos of EGCs and gastric mucosa captured using LED and laser endoscopy	LED-WLI and LED-LCI can be used to visualize EGCs with non-inferiority to Laser-WLI and Laser-LCI
Haruma et al. [68]	2022	RCT/MC	1502 patients (patients with a history of gastrointestinal cancer)	Real-time observation	The detection rate of upper gastrointestinal neoplasms tended to be higher with LCI than with WLI among patients who underwent ultra-slim endoscopy
Khurelbaatar et al. [69]	2022	Retrospective/SC	52 EGCs and 29 nonmalignant lesions	Video including cases of EGC or gastric atrophy alone using standard and ultra-thin endoscopies	Ultra-thin LCI had a higher diagnostic sensitivity and significantly higher visibility scores and color difference than standard WLI

*SC* single-center, *MC* multicenter, *RCT* randomized controlled trial, *EGC* early gastric cancer, *IM* intestinal metaplasia, *LCI* linked color imaging, *WLI* white-light imaging, *BLI* blue laser imaging, *LED* light-emitting diode, *Hp* *Helicobacter pylori*, *M-Chromo-LCI* magnifying linked color imaging with indigo carmine dye, *M-BLI* magnifying blue laser imaging, *MR* map-like redness, *IC* indigo carmine contrast method, *CI* confidence interval, *AIM* acetic acid indigo carmine mixture

**Fig. 3:**
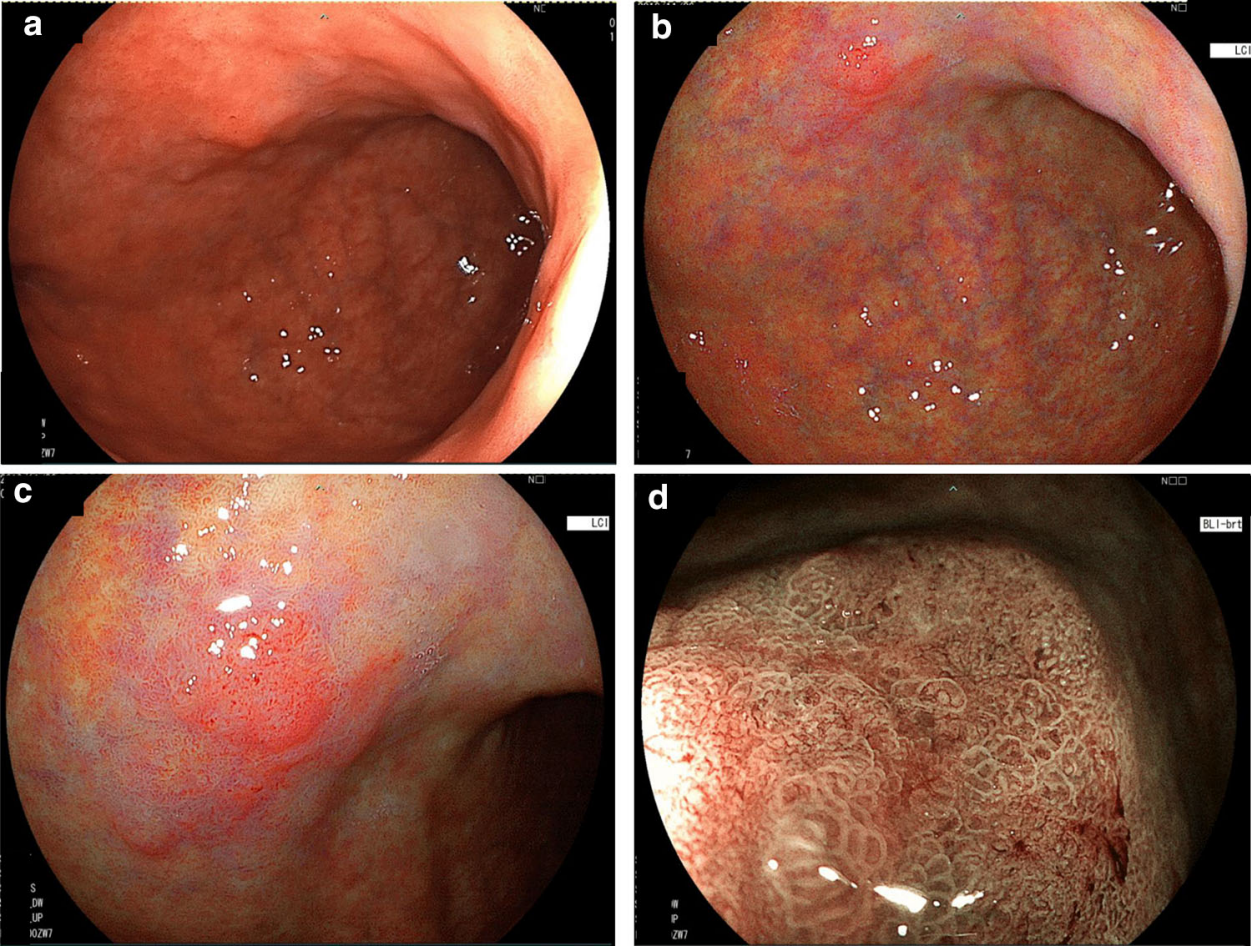
Case of early gastric cancer: flat elevated lesion. **a** Endoscopic image of the anterior wall of the gastric lower body in WLI (distant view). **b**, **c** LCI showing an orangish lesion surrounded by lavender-colored tissue (distant and close view). **d** Magnifying blue laser imaging (BLI) showing an irregular surface pattern with irregular microvasculature

Most gastric cancers are surrounded by intestinal metaplasia. The presence of widespread intestinal metaplasia makes the diagnosis of gastric cancer difficult because intestinal metaplasia can be diagnosed in a depressed area with morphological features similar to gastric cancer when using WLI. Using LCI, intestinal metaplasia is depicted in pale purple, and most of the surrounding mucosa is also depicted in purple, making it possible to obtain high color contrast [27, 46, 54]. In other words, LCI may be the most useful in cases of early-stage malignancy in which intestinal metaplasia is adjacent. EGC surrounded by intestinal metaplasia has an orange-red appearance and is surrounded by purple mucosa on LCI (Fig. 3b, c). In general, most early-stage gastric cancers are orange-red, orange, or orange-white on LCI [13]. In comparison with BLI, LCI is associated with improved visibility of EGC, regardless of the endoscopist’s experience [53, 55, 57, 58, 63, 67], and several studies have reported its advantage of improving the visibility of malignant gastrointestinal lesions with higher scores than WLI. LED-based LCI endoscopy has also been reported to be effective in detecting EGC [28, 29]. However, BLI is superior to LCI in the detection of abnormal findings in the microstructure and microvascular system of the gastric cancer due to its magnified views. Therefore, it is important to detect gastric cancer (orange) surrounded by intestinal epithelial metaplasia (purple) using LCI and to then clarify the diagnosis with magnifying BLI (Fig. 3).

A recent report showed that LCI provides more information to endoscopists and allows for the differentiated diagnosis of lesions based on mucosal color [27, 46, 50–58]. As mentioned previously, LCI shows color differences between malignant lesions and the surrounding mucosa and can increase the recognition of EGC without magnification. Some cancers with a stronger red color appear purple. These color patterns are important for early detection of gastric cancer, and a study by Yasuda et al. [63] showed the superiority of LCI over the indigo carmine contrast method and BLI in color differences between differentiated-type gastric cancer and the surrounding mucosa. A multicenter randomized controlled trial by Ono et al. [15] reported that LCI significantly increased the detection potential of neoplastic lesions of the upper gastrointestinal tract, including gastric cancer, compared with WLI. In addition, the percentage of overlooked neoplasms was lower in LCI than in WLI. Gao et al. reported that LCI had a significantly higher detection ratio of gastric neoplastic lesions than WLI [17]. Ono's and Gao's studies were performed on populations with high risk of EGC [15, 17]. In addition, the primary endpoint of Ono's study referenced by the authors is not the frequency of detected neoplasia in the stomach, but in the pharynx, esophagus, or stomach [15]. Further investigations focusing on EGC are desired to determine whether LCI is more effective in a general population for gastric cancer screening. More recently, Khurelbaatar et al. [67] showed that when compared with conventional WLI, LCI significantly improved the visibility of EGC regardless of differences in lesion morphology, histology, location, depth of invasion, and *Hp* status, which may explain the improved detection rates and reduced miss rates previously reported.

## LCI for diagnosis of EGC after eradication

Several studies have shown that eradication of *Hp* reduces not only metachronous but also primary gastric cancer [70–74]. However, gastric cancer can still develop after *Hp* eradication therapy, which is a major issue [75]. To detect gastric cancer after eradication, it is important to understand not only the cancer but also the high-risk findings of the background mucosa. Advanced intestinal metaplasia, atrophy [76, 77], and MR after eradication of *Hp* have been reported [78]. Gastric cancer detected after eradication is characterized by many difficult-to-diagnose lesions, such as the surface depression-type similar to MR with patchy redness, highly differentiated adenocarcinoma with low-grade atypia, and gastritis-like appearance [79, 80]. These characteristics are thought to be related to background mucosal changes after eradication and the phenomenon of the cancer surface layer becoming coated with normal epithelium or epithelium with low-grade atypia, similar to non-tumor mucosa [81, 82].

Reports on the detection of gastric cancer after eradication by IEE are limited. Among the gastritis findings described in the Kyoto Classification [33] of gastritis, MR is an independent risk factor for gastric cancer in both WLI and LCI, though evaluation of MR by LCI is considered better (Fig. 1c,d) [49]. The advantage of LCI in the observation of the stomach after eradication is that intestinal metaplasia and MR can be clearly observed, even at a distant view. In LCI observations, it has been reported that intestinal metaplasia is easily recognized as lavender colored, and the detection rate is significantly higher than that of WLI observations (Fig. 1a, b) [44]. After eradication, a large number of red depressions (MR) that can be seen over a large range appear in the gastric mucosa from the gastric body to the antrum. In white-light observation, the endoscopic image of these findings is very similar to that of cancer, and distinguishing between benign and malignant tumors is difficult. Furthermore, due to the variety of colors in the background mucosa, it is controversial whether the enhancement of color leads to the detection of EGC. However, in LCI, these lesions are observed in lavender color, whereas gastric cancer detected after disinfection is recognized as orange or magenta color, so it can be observed more clearly than in WLI by color contrast [62]. Moreover, Kanzaki et al. reported a more significant color difference between gastric cancer and the suspicious mucosal areas in the b* color values when using LCI than when using WLI [59]. For a definitive diagnosis, it is useful to add magnified BLI observations [83]. As previously mentioned, LCI has been reported as superior over WLI in detecting the presence of upper gastrointestinal tumors, including early-stage gastric cancer and tumorous lesions [15], which includes many gastric cancers discovered after eradication.

## LCI for diagnosis of EGC without *Hp* infection

**Fig. 4:**
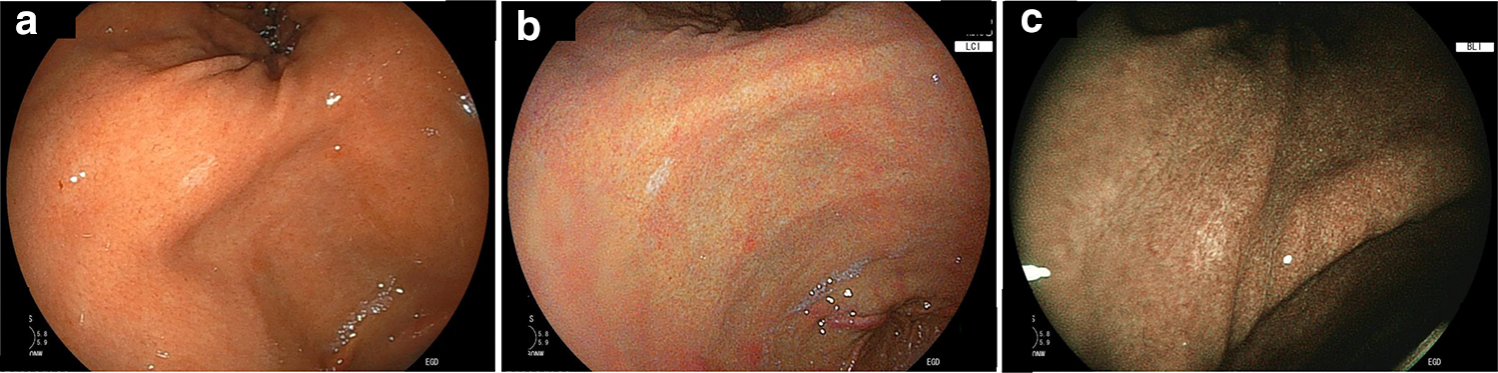
Detection of early gastric cancer without *H. pylori* infection. **a** WLI image of a signet-ring cell carcinoma of the stomach. **b** LCI image more clearly demonstrating the lesion. **c** BLI image

Conventionally, *Hp*-uninfected gastric cancer has been reported to be extremely rare, and its frequency has been reported to be approximately 1% of all gastric cancers [84, 85]. The frequency of undifferentiated-type cancer is high, and in the endoscopic classification, type 0–IIc (mostly signet ring cell carcinoma) is common [86]. Since such lesions are recognized as color changes, they are easy to detect by WLI observation; however, because the height difference between the lesion and the surrounding mucosa is poor, it can also often be unclear when dye is applied (Fig. 4). There are no data on the detection of EGC using LCI and BLI in *Hp*-uninfected gastric cancer cases. On LCI, undifferentiated gastric cancer with a faded tone, characteristic of mucosa in *Hp*-uninfected cases, can be easily observed with a clear color difference [54]. LCI cannot determine histology from endoscopic findings without enlargement; however, it may detect undifferentiated gastric cancer based on the color of the mucosa.

## Transnasal endoscopy with LCI in detecting EGC

Ultra-thin endoscopy has advanced due to improvements in technology for providing high-resolution images, and is commonly used in Japan to screen for lesions of the upper gastrointestinal tract. Recent reports have documented that ultra-thin endoscopy is equivalent to standard endoscopy [87]. Ultra-thin endoscopes can be inserted orally and nasally, thereby reducing the burden on patients undergoing endoscopy. Image-enhanced observations, such as BLI-bright and LCI, are also useful in identifying gastric neoplastic lesions with laser nasal endoscopes. Using data from the LCI-further improving neoplasm detection in upper gastrointestinal (LCI-FIND) trial, a sub-analysis showed that LCI is useful in identifying neoplastic lesions of the pharynx, esophagus, and stomach when used in ultra-slim endoscopy [68]. Khurelbaatar et al. [69] further demonstrated that ultra-thin LCI had a higher diagnostic sensitivity and significantly higher visibility scores and color differences than standard WLI. These results suggest that color contrast is more important than resolution for EGC identification. The introduction of ultra-thin LCI appears to be suitable for EGC screening in clinical practice, including routine health examinations. If lesions are visible, BLI mode is useful for diagnosing neoplastic lesions without magnification [13, 14].

## Problems of LCI for screening and diagnosis of EGC

While LCI may improve visibility of EGC in most cases when compared with WLI, it may also reduce visibility in certain cases of EGC [67]. Fukuda et al. [46] reported that malignant lesions exhibiting a redder color than the surrounding mucosa on WLI are observed as purple on LCI and may not be recognized as malignant lesions, even if the surface is irregular. This means that additional education about the significance of the purple color on LCI is required. Moreover, the endoscopic diagnosis of EGC by LCI does not have objective indicators, and interobserver agreement for LCI has been reported as fair among endoscopists in expert and non-expert groups. Therefore, it is desirable to develop an endoscopic diagnostic method that enables objective evaluation to assist in detecting lesions that is not dependent on the endoscopist’s skill. Photodynamic endoscopic diagnosis (PDED) is based on the fluorescence of photosensitizers that accumulate in tumors, which enables objective evaluation independent of the endoscopist’s experience and is useful for tumor detection [88, 89]. However, PDED is still a challenging field, and further study should be conducted to demonstrate its usefulness.

## LCI and artificial intelligence

Recently, artificial intelligence (AI) [90] using convolutional neural network-computer-aided diagnosis (CAD) systems has made remarkable progress in the field of gastrointestinal endoscopy, and it is becoming widely available and utilized for the detection of *Hp* infection status and gastric cancer [91–101]. Several studies have demonstrated the excellent ability of CAD in combination with LCI or BLI-bright to determine *Hp* infection status [97, 102, 103]. Nakashima et al. [104] reported the strong diagnostic ability of LCI-CAD to classify *Hp* infection status as follows: *Hp* positive, *Hp* negative, and eradicated *Hp*. However, there is room for further improvement in the determination of post-*Hp*-eradication status using AI.

AI allows the quick detection of early-stage gastric cancer with high sensitivity [95, 105, 106], but its positive predictive value and specificity are low (though this is rapidly improving [107]). In addition to its accuracy, AI diagnostic imaging is expected to reduce the burden of double-checking to effectively extract patients who need follow-up endoscopy [107], while ensuring quality diagnosis and support for non-expert endoscopists [108]. However, high false-negative and false-positive rates remain a major limitation of AI-assisted EGC detection. Hirasawa et al. [95] reported that most false-negative AI cases were diminutive cancers of 5 mm or less with a superficially depressed morphology. In view of this, LCI could improve the training image data of the test set by enhancing color change with brighter illumination. Therefore, AI using LCI could help reduce false-negative results and improve the quality of AI-assisted endoscopic EGC detection. In the future, the practical application of an endoscopic diagnosis support system that double-checks endoscopic images for gastric cancer screening and detects gastric cancer in real time during endoscopy is expected.

## Conclusion

In this article, we reviewed studies on the usefulness of LCI in gastric cancer screening and described the future perspectives as well as challenges of LCI in clinical practice. Compared to conventional WLI, LCI has been shown to provide excellent visibility of gastric cancer due to high color contrast relative to the surrounding tissue, thus improving the detection of lesions in distant views and facilitating the more accurate diagnosis of gastric neoplasms. Furthermore, LCI assists in the determination of *Hp* infection status, which is closely related to the risk of developing gastric cancer. In clinical practice, the use of LCI with ultra-thin endoscopy and AI-assisted endoscopy appear to be promising areas in gastric cancer screening. Future research in these areas with a focus on developing an endoscopic diagnostic method that enables objective evaluation independent of the skill of the endoscopist is warranted.

While future clinical investigations focusing on the stomach with a much larger sample of the general population are needed to further validate the routine use of LCI, we believe that LCI may play a key role in the improved detection of ECG, thus helping reduce the number of gastric cancer-related deaths.

## Data Availability

Data will be available from the corresponding author on reasonable request.
