# Correction: Current status and future perspective of linked color imaging for gastric cancer screening: a literature review

**DOI:** 10.1007/s00535-023-01971-2

**Published:** 2023-03-08

**Authors:** Kazuo Yashima, Takumi Onoyama, Hiroki Kurumi, Yohei Takeda, Akira Yoshida, Koichiro Kawaguchi, Naoyuki Yamaguchi, Hajime Isomoto

**Affiliations:** 1 Division of Gastroenterology and Nephrology, Faculty of Medicine, Tottori University, 36-1 Nishicho, Yonago, 683-8504 Japan; 2 Department of Endoscopy, Nagasaki University Hospital, Nagasaki, Japan

**Correction: J Gastroenterol (2023) 58:1–13 ** 10.1007/s00535-022-01934-z

In the original publication of the article, the “Table 1 continued” column head was wrong.

The original article has been corrected.

